# Modeling Cortisol Dynamics in the Neuro-endocrine Axis Distinguishes Normal, Depression, and Post-traumatic Stress Disorder (PTSD) in Humans

**DOI:** 10.1371/journal.pcbi.1002379

**Published:** 2012-02-16

**Authors:** K. Sriram, Maria Rodriguez-Fernandez, Francis J. Doyle

**Affiliations:** 1 Institute of Collaborative Biotechnologies, University of California, Santa Barbara, California, United States of America; 2 Department of Chemical Engineering, University of California, Santa Barbara, California, United States of America; Indiana University, United States of America

## Abstract

Cortisol, secreted in the adrenal cortex in response to stress, is an informative biomarker that distinguishes anxiety disorders such as major depression and post-traumatic stress disorder (PTSD) from normal subjects. Yehuda *et al.* proposed a hypothesis that, in humans, the hypersensitive hypothalamus-pituitary-adrenal (HPA) axis is responsible for the occurrence of differing levels of cortisol in anxiety disorders. Specifically, PTSD subjects have lower cortisol levels during the late subjective night in comparison to normal subjects, and this was assumed to occur due to strong negative feedback loops in the HPA axis. In the present work, to address this hypothesis, we modeled the cortisol dynamics using nonlinear ordinary differential equations and estimated the kinetic parameters of the model to fit the experimental data of three categories, namely, normal, depressed, and PTSD human subjects. We concatenated the subjects (n = 3) in each category and created a model subject (n = 1) without considering the patient-to-patient variability in each case. The parameters of the model for the three categories were simultaneously obtained through global optimization. Bifurcation analysis carried out with the optimized parameters exhibited two supercritical Hopf points and, for the choice of parameters, the oscillations were found to be circadian in nature. The fitted kinetic parameters indicate that PTSD subjects have a strong negative feedback loop and, as a result, the predicted oscillating cortisol levels are extremely low at the nadir in contrast to normal subjects, albeit within the endocrinologic range. We also simulated the phenotypes for each of the categories and, as observed in the clinical data of PTSD patients, the simulated cortisol levels are consistently low at the nadir, and correspondingly the negative feedback was found to be extremely strong. These results from the model support the hypothesis that high stress intensity and strong negative feedback loop may cause hypersensitive neuro-endocrine axis that results in hypocortisolemia in PTSD.

## Author Summary

PTSD is an anxiety disorder that occurs among persons exposed to a traumatic event involving life threat and injury. This is a co-morbid psychiatric disorder that occurs along with depression. Cortisol is an informative endocrine biomarker that can distinguish PTSD from other co-morbid disorders. In comparison to normal subjects, hypocortisolemia was observed during the night in PTSD, while hypercortisolemia was observed in depressed subjects. From analyzing the clinical data, Yehuda *et al.* hypothesized that hypocortisolemia in PTSD was due to the strong negative feedback loop operating in the neuroendocrine axis under severe stress. We complemented this hypothesis by constructing a mathematical model for cortisol dynamics in HPA axis and estimated the kinetic parameters that fitted the cortisol time series obtained from the clinical data of normal, depressed and PTSD patients. The parameters obtained from the simulated phenotypes also strongly support the hypothesis that, due to disruptive negative feedback loops, cortisol levels are different in normal, PTSD and depressed subjects during the night. Importantly, the model predicted the transitions from normal to various diseased states, and these transitions were shown to occur due to changes in the strength of the negative feedback loop and the stress intensity in the neuro-endocrine axis.

## Introduction

PTSD is an anxiety disorder that results from exposure to traumatic events. According to the *Diagnostic and Statistical Manual of Mental Disorders* (DSM-IV), the core symptoms are impaired concentration, emotional numbing, recurrent flashes of traumatic memories, social withdrawal, and hyperarousal [1]. Neuroendocrine studies identified the HPA axis as the site of action that brought about biochemical changes in response to severe stress and, in particular, cortisol variations were found to differ in PTSD compared to normal and depressed subjects. For the past 20 years, there were many contradictory reports about the findings of cortisol levels in PTSD that ranged from hypocortisolemia [2]–[6] to hypercortisolemia [7]–[9] to no change [10], [11], but all the reports indicated that the cortisol level was in the endocrinologic range with only subtle changes observed in neuro-psychiatric disorders. Before discussing the problem and findings of our modeling work, we provide a brief overview of various neuroendocrine findings of cortisol dynamics in PTSD patients based on the work of Yehuda [12], and discuss the corresponding hypothesis that was generated about the HPA mechanism based on the clinical data.

**Figure 1:**
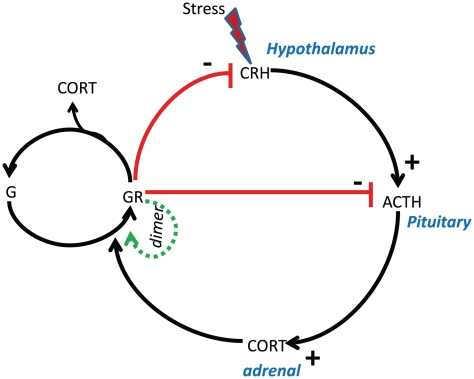
Regulatory network of cortisol in the HPA axis. Stress induces the secretion of corticotrophin release hormone (CRH) in the hypothalamus that diffuses to the pituitary gland to activate aceto-corticotrophin hormone (ACTH). ACTH in turn activates cortisol (CORT) in the adrenal gland. The secreted cortisol binds to glucocorticoid (G) receptor to form a complex GR followed by the dimerization reaction of GR complex. Cortisol down regulates its own production through GR complex that binds to both CRH and ACTH and form a closed loop. This closed loop gives rise to negative feedback in the circuit.

The stress responsive HPA axis belongs to the neuro-endocrine system that regulates cortisol through feedforward and feedback loops. Cortisol, also known as glucocorticoid, exhibits both ultradian and circadian patterns, which when disrupted result in various metabolic and psychiatric disorders including depression and PTSD. Stress induces the release of corticotrophin-releasing hormone (CRH) from the hypothalamus and activates adreno corticotrophic hormone (ACTH) in the anterior pituitary. ACTH moves to the adrenal cortex and stimulates the production of cortisol. Cortisol has a stronger affinity for mineralocorticoid receptors than for the glucocorticoid receptors (G) and forms a complex with these receptors. Both glucocorticoid and mineralocorticoid complexes undergo homo-dimerization to increase the activity of the complex [13], and GR-cortisol complex in turn binds to CRH and ACTH to down regulate the production of cortisol (see Figure 1). This negative feedback loop from cortisol is vital in maintaining the homeostasis of the system during stress. When disrupted, it results in the loss of sensitivity of the HPA axis to the negative feedback regulation, based on which the popular “glucocorticoid cascade hypothesis” was formulated [14].

In contrast to the hypercortisolism observed in depressed patients, hypocortisolism was observed in PTSD subjects. Two hypotheses were proposed for hypocortisolism in PTSD subjects; (i) enhanced negative feedback of the HPA axis, and (ii) adrenal insufficiency. The enhanced negative feedback inhibition hypothesis, first proposed by Yehuda [15], [16], explained the hypocortisolism in PTSD subjects due to hypersensitive HPA axis with a strong negative feedback loop, while in the case of subjects with major depression, the weakened negative feedback loop resulted in hypercortisolism. To support this hypothesis, Yehuda *et al.* [17] evaluated the cortisol patterns collected from human subjects screened for PTSD and major depressive disorder by carrying out chronobiological analysis that fitted the cortisol data by standard cosinor and multicosinor models, and showed that only the difference in cortisol levels at nadir was statistically significant. Even though few earlier reports contradicted this hypothesis, and no statistically significant difference in the cortisol levels was observed between normal and PTSD subjects [7]–[9], it is now widely accepted that in PTSD, the blunted cortisol secretion is due to its strong negative feedback loop. A survey of various neuro-endocrine findings on PTSD that predominately concerned cortisol dynamics was made in [18]. There are several reasons attributed to the discrepancy in cortisol levels in PTSD that include the heterogeneity of the epidemiological samples [19], [20], the methodologies used to determine cortisol levels, and the type of neuroendocrine challenges like DEX or Metyrapone tests that were used to probe the role of negative feedback loops contributed strongly to the distinction of various neuro-psychiatric disorders (see [12] and the references therein for details of the test and its outcome). However, when all these protocols were tightly maintained, it was found that in PTSD, the cortisol level during the night was much lower than normal, and this is attributed to the strong negative feedback inhibition. All these observations from negative feedback loop tests resulted in ruling out the adrenal insufficiency hypothesis.

The motivation for the present work, therefore, is to address the hypothesis proposed by Yehuda [15], since the cosinor [17] models do not have the provision to show that the differing dynamics in normal, depressed and PTSD subjects were due to the varying strengths of the negative feedback loop in the HPA axis. To show the role of the negative feedback loop requires a mechanistic model with bio-chemical kinetics, motivating the development of an endocrine model of the HPA axis. Further, mechanism based models provide insight into the role of feedback loops and the functioning of networks on the whole [21]. Specifically, the options of drug targeting and its efficacy in the treatment of these disorders can be explored, since it is known that negative feedback loops play a vital role in dampening the effects of drugs [22], [23]. Therefore, we constructed a nonlinear ordinary differential equations (ODE) model of the HPA axis to capture the 24 h cortisol dynamics of normal, depressed and PTSD subjects, and used the same data fitted by Yehuda *et al.* [17] to estimate the kinetic parameters of the ODE model through global optimization. To verify the hypothesis, two tunable kinetic parameters, namely, the strength of stress and the inhibition constant that determines the strength of the negative feedback loop were used in the model. Model construction, parameter estimation, bifurcation analysis, verification of the effect of negative feedback loops on pathology, simulation of phenotypes, summary and conclusion are elaborately explained in the following sections.

## Materials and Methods

### Assumptions, mathematical model and simulation tools

In developing the mathematical model of the cortisol molecular network depicted in Figure 1, two assumptions were made: (i) the first order dilution rate due to the transport of hormones and autonomous degradation are considered together. Apart from dilution/autonomous degradation, Michaelis-Menten kinetics are separately considered for the degradation of the hormones and hormone complexes within each specific region of the brain (hypothalamus, pituitary and adrenal); and (ii) a sufficient number of molecules is present for the reactions to take place using continuum kinetics so that stochastic fluctuations (internal noise) are eliminated. The nonlinear ODE equations for the cortisol network are framed as follows:
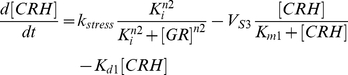
(1)

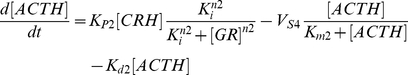
(2)

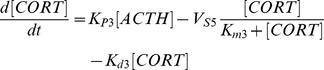
(3)

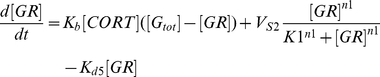
(4)

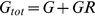
(5)CRH, ACTH, CORT, GR, and G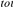 are the corticotrophin-releasing hormone, adreno-corticotrophin hormone, cortisol, glucocorticoid receptor complex, and total glucocorticoid receptor, respectively. *k* 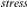 is the bifurcation parameter that drives the system by initiating the CRH production, K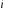 is the inhibition constant that regulates the strength of the negative feedback loop, V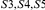 are the rates at which the hormones CRH, ACTH, and CORT are degraded enzymatically through saturation kinetics, K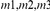 are the Michaelis constants, K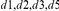 are the autonomous degradation constants, K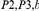 are the rates of production of ACTH, CORT, and GR respectively, *n*1, *n*2 are the Hill constants, and *K*1 is the activation constant. The negative feedback regulation by glucocorticoid receptor complex is described by Hill kinetics, the dilution/autonomous degradation rate by a first order reaction, and the enzymatic degradation by Michaelis-Menten kinetics. The glucocorticoid receptor dimerization reaction (Eqn (4)) that increases its own activity is expressed by Hill kinetics and this generates bistability in the model. The introduction of homo dimerization is akin to the model of Gupta *et al.* [24] that generates a kind of positive feedback in the model. Therefore, the present model has both a positive and a negative feedback loop, with the positive feedback loop giving rise to bistability, and the negative feedback loop giving rise to oscillations for the appropriate choice of parameters. Earlier models that described the HPA axis have either captured oscillations (both ultradian or circadian) or bistability ([25] and references therein), but the present model is capable of exhibiting both of these dynamics.

### Earlier work on the mathematical modeling of cortisol dynamics

A detailed summary of the earlier work on mathematical modeling of cortisol dynamics was recently provided by Vinthers *et al.* [25] that classified cortisol mathematical models into two types; one for the ultradian dynamics that were endogenous, and the other for circadian oscillations driven by the external light input. The mathematical models for cortisol dynamics were based on either ordinary differential equations or delay differential equations, and these models primarily explained the occurrence of ultradian rhythms [26]–[37]. In describing the dynamics of these models, two further classifications were made regarding the origin of the ultradian rhythms that occur though (i) bursting, or (ii) fixed points; i.e, through Hopf bifurcation. However, Vinthers *et al.* [25] showed that their minimal ODE model of the HPA axis was incapable of exhibiting neither ultradian nor circadian dynamics for the imposed physiological constraints on the model. Similarly, when delay was suitably introduced in their ODE model, ultradian oscillations were observed, but the experimental evidence to account for such a long time delay was absent. Together, they suggested that the present mechanisms were inadequate to model the ultradian cortisol dynamics due to some missing links, and these were suggested to be the secretion of hormones in bursts by exocytosis, or the oscillatory elimination rates. Also, various ODE and DDE models discussed in [35] indicated that autonomous oscillations were absent.

The present model consists of four dynamical variables that are inclusive of receptor dynamics. The model was formulated to exhibit only circadian oscillations that can fit the data of Yehuda *et al.* [17] for psychiatric disorders such as depression and PTSD. The main new aspect introduced in the present work is the elimination of the hormones in their respective brain regions using Michaelis-Menten kinetics that introduced implicit delay in the model, and therefore, can exhibit both ultradian and circadian oscillations. The model also has a lot of similarities to the circadian oscillatory model of Goldbeter [38], and the model of Bliss *et al* [39]. For the choice of parameters, the model can also exhibit ultradian oscillations. We have not explicitly introduced a sinusoidal term that denotes the hypothalamic drive that induces the circadian oscillations of cortisol in HPA axis, and the driving term in the present model is the constant bifurcation parameter k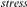. Similar to the model of Gupta *et al.* [24], we neglected the circadian drive. Apart from enzymatic degradation, autonomous degradation/dilution was also considered, and modeled using first order kinetics. Modeling the receptor complex formation, and the dimerization reaction was similar to the work of Ferrell and Xiang [40], [41]. The dimerization of receptor complex provides the positive feedback, and the dimer-cortisol complex, that binds the ACTH and CRH hormones, provides the negative feedback loop that down regulates the cortisol. The positive feedback leads to bistability, whereas the negative feedback is expected to lead to circadian oscillations for the choice of parameters.

We used the software program XPPAUT for numerical integration and to generate all the bifurcation diagrams [42]. We provide the ordinary differential equation (ODE) file of XPPAUT as a separate supplementary file. We transported the data from XPPAUT to MATLAB to plot the figures. To estimate the kinetic parameters, and to perform the sensitivity and correlation analysis, we used the toolbox SensSB [43].

### Clinical data for cortisol dynamics

Clinical data for 24 h cortisol regulation in normal, PTSD, and depressed subjects were extracted from Yehuda *et al.* [17] using the software Labnotes (V 1.0) [44]. The cortisol data from the blood was sampled at approximately 30 minutes from 10.00 AM to the next day at 10.00 AM. All the male PTSD subjects were Vietnam veterans, and the depressed subjects were outpatients with no PTSD. A distinct spike at 6.00 PM in all the three categories was observed due to the consumption of a meal, but the spike was pronounced in both normal and depressed subjects. The spiked data point was included in our analysis. Triplicate sets of cortisol data were available for the normal as well as for each of the pathological categories, and in some categories, the missing data were filled by imputation as follows; if the data of one of the three subject at one particular time point was missing, then the average value of the available data from the other two available subjects at that missing time point was taken.

**Figure 2:**
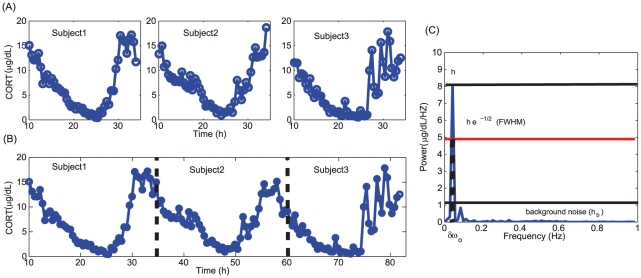
Time series and power spectrum of PTSD subjects. (A) The time series of three individual subjects, (B) concatenated time series of the three individual time series, and (C) amplitude spectrum of the time series. The height of dominant peak in the spectrum is denoted by h, and 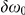 is the difference in the frequency of the time series corresponding to the full width at half maximum (FWHM, given as exp(−1/2)* *h*), 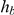 is the background noise. The fundamental frequency 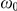 of the concatenated time series is 0.0417 Hz and this corresponds to the frequency where maximum peak “h” occur in the spectrum.

In order to estimate the kinetic parameters of the model, the time series of the three subjects were concatenated by pooling them together, and assumed to be homologous (see Text S1 for further analysis on concatenation to explain the homogeneity of individual time series). The time series were concatenated by taking the average of the last point of one subject and the first point of the next subject (Figure 2B). We concatenated the subjects (n = 3) in each category and created a model subject (n = 1) without considering the patient-to-patient variability within each case. This is done to present a model for a single patient in each group, and the variability within each of the groups is not considered due to lack of sufficient data. The signal to noise ratio of the mean subtracted concatenated time series (to remove the linear trends) was determined from the power spectrum of the time series to assess the quality of the signals. This is based on the method of Gang et al. [45], and also to verify the circadian nature of the concatenated time series (Figure 2C). The signal-to-noise ratio (SNR) was determined using the expression *h* 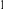 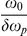, where *h* 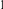 is the ratio of the height of the amplitude spectrum (*h*) to the background noise (*h* 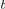), which is is the sum of the background noise obtained between 0–1 Hz (half of the sampling frequency, 2 Hz), 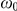 is the frequency of the spectrum corresponding to the maximum amplitude and 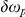 is the frequency difference at full width half maximum (see Figure 2C). The ratio 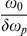 is known as the quality/regularity factor *q* of the signal. A large SNR value indicates a strong signal-to-noise ratio [45], and this was used to determine the degree of coherence of the signal in the presence of noise.

The frequency of normal, PTSD, and depressed subjects' concatenated time series was found to be 0.0417 Hz, which was close to the circadian period. The SNR for normal, PTSD, and depressed subjects were 5.3, 11.7, and 15.5, respectively. These values indicated that PTSD signal has a good SNR, while the concatenated time series of depressed subjects has a lower SNR than PTSD. This is in accordance to the trend observed by Yehuda *et al.* [17] that in the case of depressed patients, the weakened negative feedback loop in the HPA axis resulted in a noisy time series with poor SNR. Also, it is well known that a strong negative feedback loop provides good homeostasis, and low fluctuations in the system [46], and the SNR values indirectly support the view that a strong fluctuation in depressed patients may be due to a weakened negative feedback loop. In the next section, it is shown through model simulation that depressed subjects indeed have a weakened negative feedback loop.

## Results

### Parameter estimation

The nonlinearities of the model led to a multimodal parameter estimation problem, and therefore required global optimization methods to avoid convergence to local solutions [47]. In this work we used the algorithm SSm, available in the SensSB toolbox, that has been shown to be a powerful metaheuristic for kinetic parameter estimation of biological ODE models [48]. Although more parameters could be different between the three groups, according to the hypothesis, only two kinetic parameters, namely 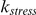 and 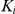, are considered to be significantly different in the three pathological cases. Therefore, the model calibration was performed simultaneously for the three time series, allowing 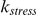 and 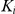 to differ for all the three cases, and forcing the remaining 18 parameters to be the same. The cost function was defined as the weighted least squares function (*J* 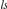) resulting from the sum of the squared distances between the experimental and predicted values of cortisol at each of the sampling points for normal, depressed, and PTSD, and it is given as follows:
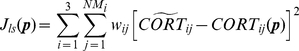
(6)where 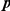 is the vector of parameters, 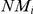 is the number of measures for the experiment 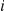 (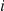 = 1, 2, 3 for Normal, Depressed and PTSD, respectively), 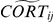 is the experimental value of the cortisol for the experiment 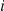 at the sampling point 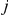, and 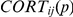 is the corresponding value predicted by the model. Since both the peak and the nadir of the cortisol concentration are critical in our study, the weights we chose were 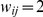 for the 10% highest and 10% lowest experimental data, and 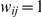 for the rest of them. Moreover, a death penalty constraint enforces the system to oscillate with a period close to 24 hours in order to agree with the circadian oscillations of the experimental cortisol data, and the kinetic parameters that led to stable steady states were discarded. For each iteration of the optimization method, the model was simulated until sustained limit cycle oscillations were achieved after discarding the transients, and the maximum value of the oscillation was taken as the first point to fit the experimental data for all the three categories of the concatenated time series.

**Figure 3:**
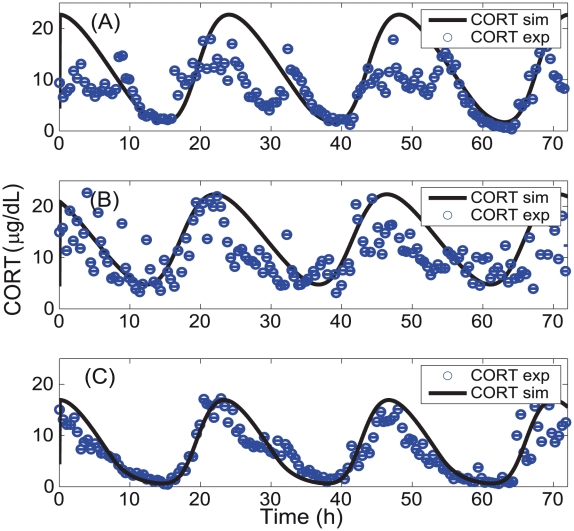
Experimental and fitted concatenated cortisol time series from the model. (A) Normal (B) depression, and (C) PTSD. The parameter set used to simulate each of the category is given in Table 1.

**Figure 4:**
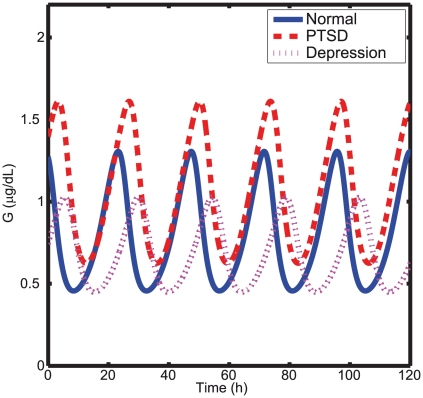
Simulated free glucocorticoid receptor time series of normal, depressed, and PTSD subjects. The free glucocorticoid receptor (G) in PTSD is much higher than in normal and depressed subjects indicating a stronger negative feedback loop.

A good agreement between the experimental and the model predicted time series for all the three subjects was obtained (Figure 3) although the fit for the depressed (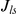 = 69.2) and the PTSD patients (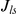 = 36.9) was much better than for the normal patients (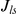 = 82.9). Importantly, the model captured the significantly reduced levels of basal cortisol for PTSD subjects. Further, the free glucocorticoid-receptor concentration (see Figure 4) was found to be higher for the PTSD than for the normal subjects, which agrees with Yehuda's findings [16], [49]. Even though glucocorticoid-receptor concentration was not used in the model fitting, its elevated simulated level in PTSD reinforces the predictive power of the present model.

The estimated strength of the stress determined by the parameter 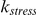 was higher for PTSD than for both depressed and normal subjects (see Table 1), and this was expected for an anxiety disorder precipitated by extreme stress. On the other hand, the estimated inhibition constant 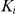 value was much smaller for PTSD than for both normal and depressed subjects, indicating the presence of stronger negative feedback loop. These values support Yehuda's hypothesis suggesting that an enhanced negative feedback of the HPA axis is responsible for the hypocortisolism observed in PTSD subjects, and this result explains the apparent contradiction of low cortisol levels in a disorder precipitated by extreme stress.

**Table 1:** Estimated kinetic parameters of normal, PTSD, and depressed subjects used in the bifurcation analysis and numerical integration.

Constants	Literature values	Source and Ref	Lb	Ub	Optimized values
*k* 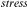 (Normal)	0.76 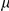 M 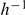	vs [19]	5	20	10.1 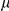 g dL 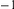 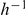
*k* 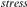 (Depressed)	0.76 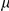 M 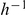	vs [19]	5	20	13.7 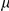 g dL 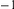 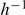
*k* 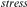 (PTSD)	0.76 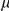 M 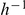	vs [19]	5	20	17.5 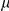 g dL 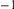 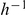
*k* 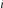 (Normal)	1 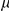 M	k1 [19]	0.5	3	1.51 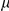 g dL 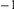
*k* 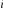 (Depressed)	1 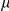 M	k1 [19]	0.5	3	1.60 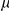 g dL 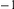
*k* 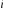 (PTSD)	1 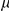 M	k1 [19]	0.5	3	1.17 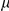 g dL 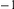
*V* 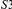	1.58–5 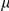 M 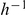	v1–v4 [19]	3	4	3.25 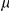 g dL 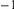 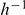
*K* 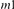	2 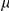 M	k1–k4 [19]	1	2	1.74 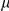 g dL 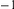
*K* 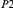	0.3–1.8 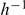	ks/k1 [19]	7	11	8.30 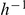
*V* 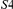	1.58–5 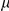 M 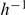	v1–v4 [19]	0.5	1.5	0.907 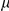 g dL 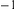 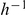
*K* 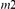	2 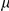 M	k1–k4 [19]	0.08	2	0.112 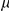 g dL 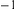
*K* 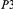	0.3–1.8 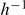	ks/k1 [19]	0.5	1.2	0.945 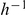
*V* 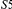	1.58–5 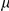 M 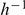	v1–v4 [19]	0.001	0.008	0.00535 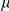 g dL 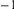 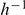
*K* 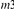	2 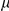 M	k1–k4 [19]	0.03	0.08	0.0768 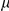 g dL 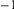
*K* 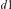	0.173 min 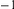	CRH degradation [28]	0.002	0.005	0.00379 h 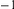
*K* 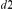	0.035 min 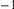	ACTH degradation [28]	0.001	0.01	0.00916 h 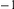
*K* 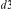	0.009 min 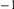	CORT degradation [28]	0.1	0.5	0.356 h 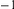
*n1*	5	n [41], [42]	4	6	5.43
*n2*	4	assumed	4	6	5.10
*K* 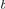	-	[41], [42]	0.008	0.05	0.0202 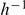
*G* 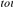	not known	assumed	2	5	3.28 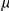 g
*V* 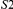	0–1 (arb.units)	vs2 [41], [42]	0.01	0.07	0.0509 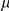 g dL 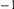 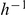
*K1*	1 (arb.units)	k1 [41], [42]	0.2	0.7	0.645 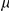 g dL 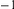
*K* 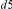	0.01 (arb.units)	kd5 [41], [42]	0.04	0.09	0.0854 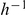

Lb and Ub are the lower and upper bounds respectively used after multiple iterations. The Hills coefficient “n” is taken as 4 because the 2 molecules of cortisol binds to the dimer to form a complex that is involved in the negative feedback regulation. *k* 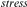 is the estimated bifurcation parameter used in the simulation of the full ordinary differential equation (ODE) model. To simulate the bifurcation diagram, the estimated *k* 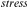 for normal, PTSD and depressed subjects was adjusted to reach the stable steady state, after which the parameter was slowly increased to sweep the region for bistablity and Hopf bifurcation. All the other parameters employed for bifurcation analysis were kept constant. The degradation rates *K* 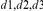 are extremely high to be directly used in the simulations. Therefore, we have chosen a much smaller lower and upper bound values for all the degradation values by scaling down all values in hours approximately by a factor of 100–1000.

**Figure 5:**
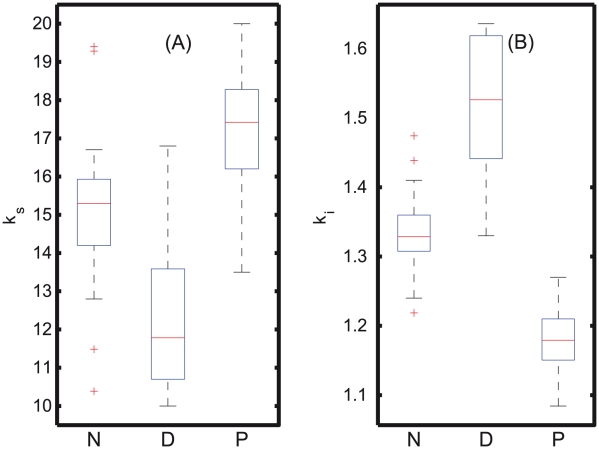
Whisker plots for stress and inhibition strength parameters of the 50 phenotypes. (A) The reestimated stress parameter of the 50 sets indicates that for PTSD (k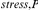) is much higher than for depressed (k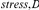) and normal (k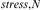) subjects. (B) The fifty parameters K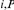 obtained for PTSD are lower than these for normal (K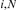) and depressed subjects (K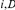), indicating the presence of a strong feedback loop. On the other hand, the depressed patients have scattered, and larger K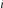 values indicating a disruptive, weak negative feedback loop in comparison to the normal subjects.

**Figure 6:**
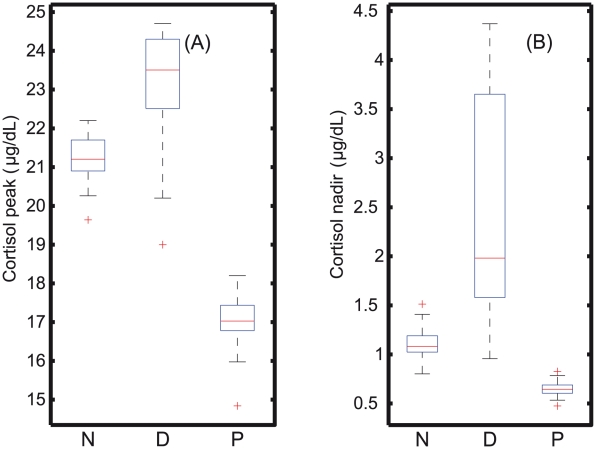
Whisker plots of the peak and nadir cortisol levels of the 50 phenotypes. (A) Peak and (B) nadir levels of the cortisol for the normal (N), PTSD (P), and depressed (D) categories, for which significant group differences are observed (p-value
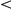
0.05). For depression, there is a wide range of cortisol values observed at nadir, whereas in PTSD, the range is extremely small, indicating that in depression, due to the weak negative feedback, the fluctuations are found to be enormous that resulted in a wide range of cortisol values.

In order to assess the statistical significance of this finding, the parameters k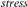 and K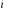 were reestimated 50 times keeping all the other parameters constant. Due to the high correlation between these two parameters and the noise in the measurements, different values were obtained in each optimization, even though a global optimization method was used. The different k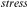 and K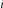 values for 50 optimizations are shown in Figure 5A–B, and the Kolmogorov-Smirnoff (KS) test indicates a significant difference between normal (N), PTSD (P), and depressed (D) sets (p-value
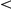
0.05). It is interesting to note that the feedback strength for PTSD was consistently much stronger (lower K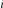 values) than for depression (larger K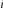 values) as expected from the hypothesis. The peak and nadir cortisol concentration for the 50 sets of parameters were also determined as shown in Figure 6A–B. KS-test indicates a significant group difference between normal (N), depression (D), and PTSD (P) (p-values
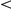
0.05). In tandem with the clinical data [17], the nadir values were significantly different between N-P, N-D, and D-P. Also, it was observed that the simulated nadir for PTSD had very little variations within the different sets of parameters found, while simulations corresponding to depressed subjects showed very large variations in comparison to normal subjects, and this was due to the difference in the strength of the negative feedback loops. The only discrepancy from the clinical data was that, the simulated peak values for normal (N) and PTSD (P) were found to be significantly different (p-values
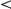
0.05). Despite this discrepancy, the simulation of the reestimated parameter sets supports the hypothesis that PTSD arises due to the strong stress and strong negative feedback that results in hypocortisolemia from early night through late morning, whereas in depressed patients, the negative feedback is completely weakened and results in hypercortisolemia in comparison to normal subjects.

### Sensitivity and correlation analysis

To gain further insight into the cortisol dynamics predicted by the model, a local sensitivity analysis was performed for the optimal set of parameters given in the Table 1. Parametric sensitivity analysis aims to investigate the effect of parameter changes on the model output, in this case, the concentration of cortisol. In this study we used relative sensitivity indices, computed by multiplying the partial derivative (the absolute sensitivity function) by the nominal value of the input and dividing by the output value. The relative sensitivity index (SI) of the model output 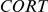 to variations in the parameter 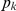 evaluated for the optimal set of parameters 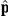 is given by:
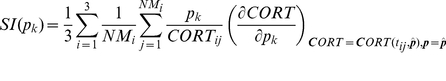
(7)


**Figure 7:**
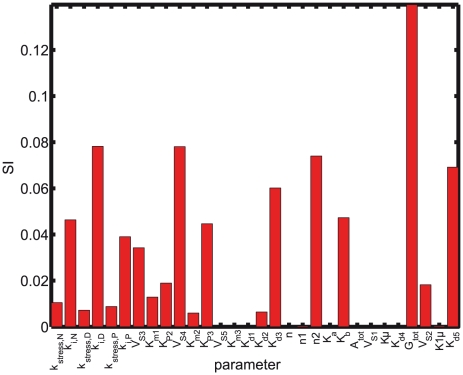
Sensitivity Indices (SI) for the optimal set of parameters. Sensitivity analysis shows that the sensitivity of cortisol with respect to K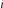 for depressed subjects is much higher than for normal subjects, while for PTSD is much lower. This difference is due to the difference in the sensitivity of the HPA axis for normal, depressed, and PTSD subjects. On the other hand, hardly any difference in the sensitivity to k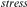 is seen. No sensitivity to the Hill coefficient n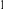 is observed, which is the homodimerization of GR receptor, indicating that the circadian oscillations are due to the other Hill coefficient n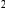, that determines the nonlinearity and robustness of the oscillations. The most sensitive parameter is the total glucocorticoid receptor, which plays a strong role in cortisol feedback regulation. This is in tandem with the hypothesis. The insensitive parameters V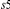, K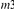, are the enzymatic degradation of the cortisol, while K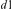 is the autonomous/dilution rate of the CRH. This indicates that in the model cortisol enzymatic degradation is not necessary, because of the strong binding rates (k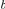 highly sensitive) of the GR receptors and, the autonomous degradation k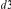 (again highly sensitive), that removes the cortisol much more efficiently than the enzymatic degradation.

Robustness and sensitivity are “two sides of the same coin” [50]–[52]. The cortisol concentration for depressed subjects was found to be the most sensitive to changes in the inhibition constant 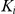 among the three groups (see Figure 7). In contrast, due to the stronger negative feedback loop, the cortisol sensitivity to 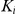 for PTSD subjects was much smaller than for normal and depressed subjects reinforcing the notion that strong negative feedback loops confer robustness to biological systems.

**Figure 8:**
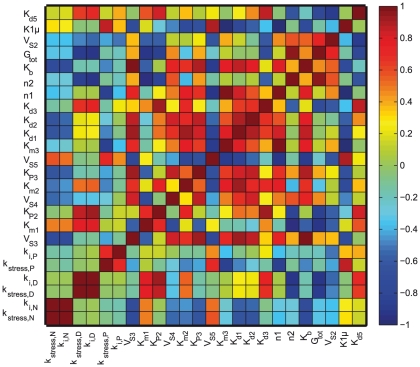
Correlation matrix. The correlation values between the dynamic sensitivities for the all the parameters are shown with the diagonal being self correlated. The levels of correlation are differently shaded as shown on the horizonal bar on the right that ranges from highly correlated (+1) to anti-correlated (−1). The sensitivities of the parameters of interest, namely k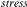 and K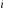 are shown to be strongly and positively correlated, whereas the sensitivity of G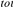, the total glucocorticoid is strongly anti-correlated with respect K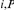, the strength of negative feedback loop in PTSD.

The correlation between parameters was studied by computing the correlation between the dynamic sensitivities as described in [53], [54]. The correlation matrix (see Figure 8) showed a strong positive correlation between the sensitivities of the parameters 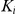 and 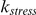. This can be interpreted as the “compensatory effect of feedback to stress” with a low value of 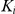 needed to maintain cortisol levels at an endocrinologic range (although lower than the normal as reported for the majority of PTSD subjects [12]) when the stress 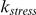 is high.

The autonomous degradation constant for the cortisol (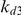) was found to be two orders of magnitude higher than the rate for enzymatic degradation 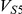 indicating that the autonomous degradation of cortisol dominates the degradation process. Accordingly, the sensitivity analysis confirmed that the model is almost irresponsive to changes on parameters 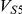 and 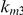, but highly sensitive to changes in 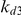. In the case of CRH, the opposite effect was found; the autonomous degradation constant 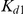 is smaller than the enzymatic degradation rate 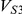 leading to a very low sensitivity for 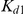 and a high sensitivity for both 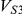 and 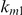. This suggests that the enzymatic degradation plays a more prominent role than the autonomous degradation for CRH. Similar behavior was found for the degradation of ACTH. The low sensitivity of the degradation rates of CRH and ACTH (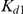 and 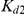) can also explain that the estimated values are far from the values reported in the literature since changes in these values would not make a big difference in the cortisol dynamics. However, a very high correlation was observed between the pairs of parameters 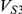-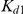 and 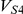-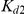, suggesting that other phenotypes with high autonomous degradation and low enzymatic degradation leading to similar cortisol dynamics could possibly be found.

### Bifurcation analysis: modulation of Hopf point with changes in negative feedback strength

**Figure 9:**
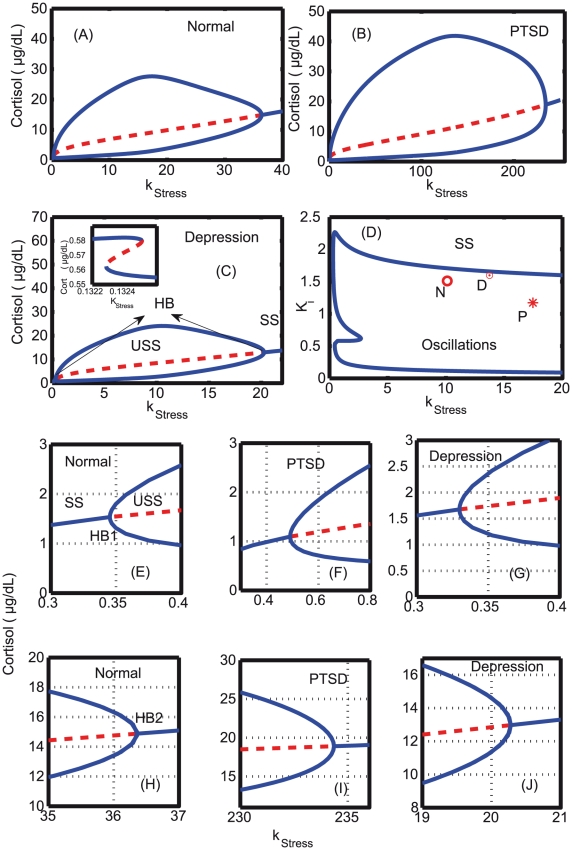
One and two parameter bifurcation analyses. Bifurcation analysis is carried out for (A) normal, (B) PTSD, and (C) depressed subjects with k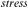 as the bifurcation parameter. All the parameters were estimated, and kept constant throughout the simulation except for k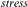 and K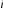 values that are differed in the normal, depressed and PTSD subjects. The parameters are given in Table 1. Initially, for simulating the bifurcation diagram, k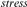 was chosen as 0.001, a stable steady state. As k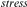 increased, bistability was only observed in depressed patients (shown as inset in (C)), and Hopf bifurcation (indicated as HB) was observed in all the three subjects. Also, Hopf bifurcation began (E-G) and ended (H-J) at different k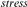 values in all the three subjects. (D) Two parameter bifurcation diagram indicates the presence of oscillations for a wide range of k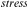-K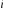 values, and N (circle), D (dotted circle) and P (star) indicates the estimated parameters for which normal, depressed and PTSD subjects lie in the parameter plane. SS–stable steady state, USS–unstable steady state, and HB–Hopf bifurcation which is supercritical in all the subjects.

**Figure 10:**
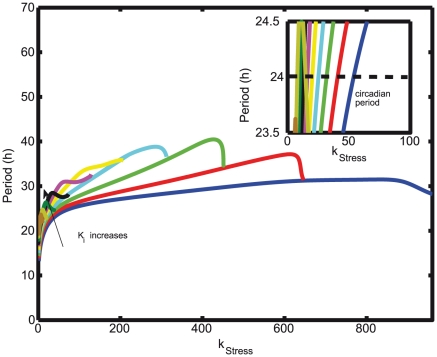
Variation of period to the corresponding changes in stress and inhibition strength. The inhibition strength K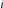 is varied from the lowest (0.8) to the highest value (1.7) in steps of 0.1 along the direction indicated by the arrow, and the bifurcation parameter is k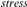. Inset shows the period of cortisol dynamics that varies between 23.5 and 24.5 for a very wide range of k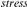 values indicating the robustness of the system.

Bifurcation analyses were carried out by varying *k* 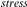, the intensity of stress, as the bifurcation parameter and cortisol as the dynamical variable. The estimated parameters for normal, PTSD, and depression were used to perform the analyses, except for *k* 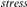 that was tuned to determine the occurrence of Hopf points. Bifurcation diagrams for normal, PTSD and depressed subjects were separately constructed as shown in Figure 9, and all the three categories exhibit both bistability and Hopf bifurcation. As stress was increased from a low value, a z-shaped saddle-node bifurcation was observed. This can be interpreted as the time immediately aftermath of the trauma during which the cortisol level decreases as stress increases with a transition from hyper to hypocortisolemia. Hypocortisolemia is captured by the z-shaped bistablity in the model. This we attribute due to “peri/acute-trauma”. Evidences from the motor vehicle accident survivors suggest that their cortisol level immediately following the accident was significantly lower, and these survivors met the criteria for PTSD [55], [56]. This was followed by a supercritical Hopf bifurcation with an unstable steady state surrounded by a stable limit cycle [57]. There are two such Hopf bifurcations found in the system, and both are supercritical in nature. The first Hopf point, HB1, was found at a lower *k* 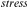 value, and the other Hopf point, HB2, terminated at a high *k* 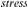 value. The bifurcation diagram indicates that for the choice of parameters obtained from the fitted data, the Hopf points are different for each of the categories. The birth of the Hopf points are advanced in the depressed patients, and delayed in the case of PTSD in comparison to the normal patients as shown in Figure 9 (E–G). Similarly the Hopf point terminates at a much lower *k* 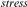 value in the depressed subjects, and at an extremely high value in PTSD subjects in comparison to the normal subjects (H–J). All the parameters in the simulation of bifurcations were the same except for the negative feedback strength, K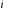, that was different in all the three categories. This indicated clearly that the stronger feedback in PTSD delays the Hopf bifurcation, but provides a very robust and wide bifurcation regime. Two parameter bifurcation analysis was also carried out in the *k* 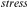
- *K* 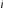 parameter plane to map out the oscillatory regimes for all the three different subjects. The wide oscillatory regime found in the *k* 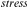-K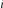 plane indicates the robustness of the oscillations to the k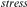-K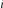 parameter changes. From the estimated parameters, the depressed subjects (shown as 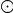) were found close to the boundary between oscillations and steady state, PTSD (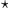) were found inside the oscillatory region, and the normal (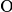) were found below the depressed category (Figure 9D). The period of the oscillations for the parameter values that were obtained from the estimation is close to circadian, and a very wide variation of the period to changes in *k* 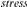 and K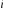 indicates the robustness of the oscillatory period to parameter changes (Figure 10).

### Predictions of pathological transitions

**Figure 11:**
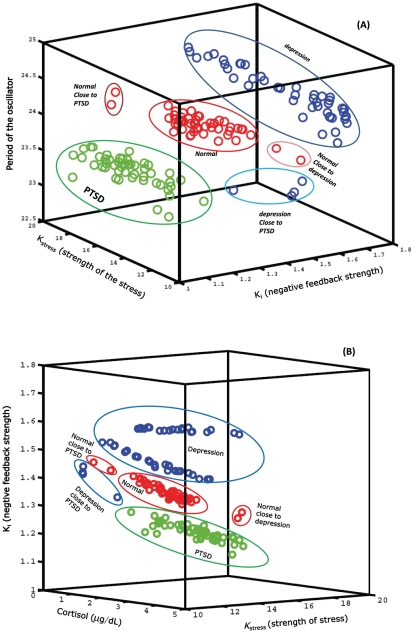
Distinction of normal, depressed and PTSD phenotypes. (A) Normal, depressed and PTSD patients are distinguished in the stress-feedback-period parameter space. All three categories can be clearly distinguished with PTSD having a strong negative feedback (low K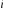 values). (B) Normal, depressed, and PTSD patients are distinguished in the stress-feedback-cortisol (nadir) concentration space. The cortisol concentration is found to be lower for PTSD, with low K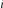 (strong feedback) values in comparison to the normal. In (A) and (B), clearly the transition from Normal 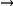 PTSD (moving towards low K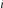 in (A), low cortisol values in (B)), Normal 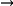 Depression (moving towards high K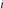 values, high cortisol values), and Depression 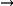 PTSD (moving towards a low K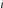, low cortisol values) can also be distinguished. PTSD 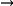 depression (moving towards a high K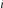, high cortisol values) can also be distinguished.

Normal, depressed and PTSD patients are also clearly distinguished in the stress-feedback-period parameter space (see Figure 11A) and in the stress-feedback-cortisol parameter space (see Figure 11B). PTSD is a co-morbid disorder that may occur along with depression. There are two possible ways for the transition from normality to a disease state can take place: (i) normal 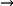 PTSD 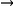 depression, (ii) normal 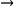 PTSD and the simulation predicts these transitions. There are also intermittent or borderline cases where the normal subjects may possibly transit to PTSD, which, when left untreated, may further degenerate to depression. Large population of individuals with PTSD share also major depression, and it has been observed that PTSD and depression share 10 out of 17 symptoms on the Hamilton rate of depression [58], [59]. All these possibilities were captured in the model as the variations in the stress-feedback and strength-period parameter space. As indicated by the hypothesis, in the parameter space, PTSD has a low nadir cortisol value (hypocortisolemia) due to the strong negative feedback loop (lower K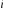 lower in the feedback loop), while depression has a elevated nadir value (hypercortisolemia) due to a weakened negative feedback loop.

## Discussion

PTSD is a neuro-psychiatric disorder that requires prevention and intervention, for which identifying biomarkers is of paramount importance. The question that cortisol can be considered as a suitable biomarker for the psychiatric disorders is debatable, since the cortisol levels of PTSD and depressed patients are present within the normal endocrinological range. The problem is further compounded by the fact that most of these disorders are co-morbid in nature and cannot be clearly distinguished whether the subjects are both PTSD and depressed or either one of them. However, according to the hypothesis, the strength of stress and the negative feedback loops in the HPA axis are different for PTSD and depression, and capturing this difference through the model constitutes a substantial part of the present work.

Mathematical modeling of cortisol dynamics have been carried out in previous studies, yet new models have been formulated and refined based on the information obtained from recent molecular biological techniques that further provided insight about the regulation and functioning of the network. There are multiple models describing the cortisol ultradian dynamics, but hardly any model was built towards understanding of hypocortisolemia in psychiatric disorders except that of Gupta *et al.* [24], who modeled the chronic fatigue syndrome that resulted in hypocortisolemia. Their dynamical model distinguished the normal and pathological states as two stable steady states (bistable, saddle-node bifurcation) separated by an unstable steady state, and the two stable steady states can be traversed through by varying the strength of stress and initial conditions. The bistable dynamics in their model were due to the homo-dimerization of the glucocorticoid receptor-cortisol complexes, but the effect of the negative feedback loop, which was conspicuous in the HPA axis, its role in hypocortisolemia, and the generation of circadian/ultradian oscillations were not fully explored. The cortisol was not modeled for its oscillatory dynamics, and the rise and fall of cortisol in their model was obtained by modulating the stress bifurcation parameter as square wave dynamics that behaved more like an excitable system. The present model which exhibits both bistability and oscillations, emphasizes the importance of negative regulatory feedback loops that strongly up or down regulate the cortisol production in a circadian fashion in various neuro-psychiatric disorders. The model was built specifically to explain the role of negative feedback loops that can distinguish depressed and PTSD from normal subjects. In contrast to the models discussed in [35], the present model also indicates that strong nonlinearity in the form of Hill's function and the implicit delay introduced through Michaelis-Menten kinetics generates intrinsic oscillations in the HPA axis without an external periodic forcing. This is one aspect that is introduced in the present work but requires further investigation. However, one drawback of the model is that the concentration of both CRH and ACTH exceeds the endocrinologic range as in most of the earlier published models for ultradian oscillations [30]. This happens despite the fact that enzymatic degradation was considered along with autonomous degradation as indicated in [25]. Also, while fitting the data for cortisol, corresponding ACTH and CRH circadian oscillatory data were not available, and therefore, the range for ACTH and CRH concentrations could not be exactly calibrated. Importantly, the limitation of the present study is that we have concatenated the subjects (n = 3) in each of the category to single model subject (n = 1) by assuming the homogeneity of subjects, and neglecting the patient-to-patient variability. So this can only be considered as an initial theoretical study, and the requires more data to validate these findings.

The mechanistic clue that negative feedback loop in cortisol may determine different psychiatric disorders was first proposed by Yehuda, and this modeling study validates that hypothesis in a quantitative framework. However, the model contradicts the view of Yehuda that cortisol levels are insignificantly different at the peak, and this may be possibly due to the simplification, or some missing links in the model as suggested by Vinthers *et al.* [25]. Finally, the model is able to predict the transition between different disorders and it proposes that the transitions can be controlled by reducing the stress and regulating the strength of the feedback loop. This result is in tandem with an earlier study in which it was showed that a mild augmented cortisol treatment regimen followed for the PTSD patients with hypocortisolemia have reduced retrieval of excessive traumatic memories [60]. However, the exact mechanistic details about the role of cortisol negative feedback loop in the treatment were not clear. Therefore, it may be interesting to analyze the strength of negative feedback loop in this regimen by performing DEX or Metyrapone tests [12]. In summary, our model supports the view that the hypocortisolemia in PTSD is due to a strong negative feedback loop as hypothesized, and that the weak negative feedback loop is responsible for depression. Hypocortisolemia in the model was observed at all the time, and predicted statistically significant differences in cortisol levels even during the day (i.e., peak levels) among normal, PTSD and depressed subjects for the concatenated patients. Again, more data is needed to validate this conclusion that is arrived with a very sparse amount of data. Importantly, the model predicted that the transitions from normal to PTSD, and vice-versa, PTSD to depression, and vice-versa, were due to a disrupted negative feedback loop, which when suitably regulated, can treat the disease.

## Supporting Information

Figure S1 **Individual and concatenated time series, power spectrum of the cortisol.** Individual 24 h time series of the PTSD, normal, and depressed time series are shown on the left column. The concatenated time series for 72 h is shown in the middle column. In the last column the amplitude spectrum for the concatenated time series is shown. The amplitude spectrum in all the three cases indicates a strong, dominant 24 h (0.0417 Hz) peaks, but there is also a peak with periodicity of approximately 16 h (0.0625 Hz). This may be due to sudden spike in the cortisol due to the consumption of a meal. The other peak is around 0.0834 Hz, the first harmonic of the fundamental peak.
(EPS)

Figure S2
**Autocorrelation, cross-correlation, instantaneous phase, and phase difference of the normal, depressed and PTSD times series.** The autocorrelation of the individual time series of the normal, depressed, and PTSD indicates only small differences in the lag (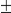
2 h from the zero lag) among the subjects. Again only small differences is seen in the cross correlation among the individual subjects in each of the categories indicating that three subjects have the small differences in the time lags (at 0
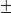
2 h). Instantaneous phase and phase difference in the right column are shown in the down right column (Note: The phase is unwrapped). In normal, large phase difference is seen for subject 3.(EPS)

Text S1 **Autocorrelation, cross correlation and phase of the individual and concatenated time series.** We provide here the justification of the concatenation of 3 patients time series.
(PDF)
