# Supplementary material for: Modeling Cortisol Dynamics in the Neuro-endocrine Axis Distinguishes Normal, Depression, and Post-traumatic Stress Disorder (PTSD) in Humans
Source: PLoS Comput Biol. 2012 Feb 16;8(2):e1002379. doi: 10.1371/journal.pcbi.1002379 (PMC3280965; doi:10.1371/journal.pcbi.1002379)
Supplement: Text S1 — Autocorrelation, cross correlation and phase of the individual and concatenated time series. We provide here the justification of the concatenation of 3 patients time series. (PDF) [file pcbi.1002379.s003.pdf]

Text S1: Autocorrelation, cross correlation and phase of the individual and concatenated time series

We provide here the justification of the concatenation of 3 patients time series. The justification for the homogeneity assumptions of the time series is supported by (a) the experimental data and the corresponding analysis carried out in [1], and (b) the time series analysis carried out for the individual and the concatenated data based on the idea in [2].

From Yehuda's result [1], the time series collected from the PTSD patients are based on the clinically administered PTSD scores (DSM III-R criteria), and the cortisol data collected from the PTSD population are distinguished from the depressed (scored using Hamilton depression rating) and the normal (scored based on medical history and laboratory screens) populations. The details of the scoring and the criteria for distinguishing normal, depressed, and PTSD patients are given in the methods section of [1]. Statistical analysis of the cortisol levels in normal, PTSD and depressed population at different time points in 24h time series in [1] were shown to be significantly different by repeated two way ANOVA measures. Multi-cosinor analyses in [1] also revealed a significant group difference between normal, depressed and PTSD patients. Therefore, the patients grouped in normal, depressed, and PTSD subjects were all different, but within each group the cortisol secretions of the patients were the same. Since the statistical analysis indicating group differences has already been established clearly in [1], we have not further proceeded in this direction.

To determine the circadian periodicity and phase, we carried out the time series analysis of individual and concatenated time series along the lines suggested in [2]. In Figure S1 all the concatenated time series exhibits a strong 24h dominant peak, but a strong ultradian peak at 16h is observed in the normal patients, presumably due to a consumption of a meal. Having established a strong 24h dominant circadian peak, the homogeneity of the normal, PTSD and depressed patients are determined using the autocorrelation and cross-correlation analysis.

The autocorrelation function (ACF) is carried out for the individual and concatenated time series, and the cross-correlation analysis is carried out between the individual time series (Figure S2) (xcorr function in MATLAB is used to determine correlation coefficients of mean subtracted time series). The autocorrelation and cross-correlation coefficients for the normal, depressed and PTSD subjects shows that there are only small inter-individual variability in each of the groups ($\pm$ 2h shift from the zero lag). The ACF values falls to zero level in all the cases for a lag of approximately 10h and crosses the zero again to rise up to the maximum of close to 24h. The concatenated time series also indicates the same. The cross-correlation in each of the category has a symmetrical bell shape pattern with a maximum occurring $\pm$2h shifted from the zero lag. Autocorrelation and cross-correlation indicates that there are only small differences in the three patients in each of the categories with no large inter individual differences, indicating that the homogeneity of the subjects in each of the categories can be assumed. Instantaneous phase and phase differences among the individual subjects in each of the categories are also calculated using Hilbert transform (Note: The phase is unwrapped). For PTSD, no difference in phases and phase differences are seen, while in depression a constant phase difference ($\sim$ 10-15° radians) is seen due to noise, and in normal, subject 3 differed largely from subjects 1 and 2 (see Figure 2). Except for one subject in normal, the instantaneous phases are close to each other in all the categories. Since very small intra individual variabilities are seen in each of the categories, homogeneity of the subjects are assumed for the concatenation of the individual time series in each of the categories.

Finally, it should be noted that due to the small sample size and limited length of the time series data, patient-to-patient variability cannot be conclusively determined.

References

1. Yehuda R, Teicher M, Trestman R, Levengood R, Siever L (1996) Cortisol regulation in posttraumatic stress disorder and major depression: A chronobiological analysis. Biol Psychiatry 40: 79-88.

2. Vagnucci A, Wong A, Liu T (1974) Time series analysis of hormonal patterns in human plasma. Comput Biomed Res 7: 513-532.
